# Muscular grip strength normative values for a Korean population from the Korea National Health and Nutrition Examination Survey, 2014–2015

**DOI:** 10.1371/journal.pone.0201275

**Published:** 2018-08-20

**Authors:** Miji Kim, Chang Won Won, Maengkyu Kim

**Affiliations:** 1 Department of Biomedical Science and Technology, College of Medicine, East-West Medical Research Institute, Kyung Hee University, Seoul, Korea; 2 Department of Family Medicine, College of Medicine, Kyung Hee University, Seoul, Korea; 3 Department of Physical Education, Kyungpook National University, Daegu, Korea; Public Library of Science, UNITED KINGDOM

## Abstract

**Introduction:**

Muscle weakness is linked to a range of adverse health outcomes across the lifespan including mortality, morbidity, and disability. Because lifestyles and body composition are quite different between Western and Asian countries, there is an urgent need to establish normative grip strength values for individuals of Asian descent. This study presents normative data for hand grip strength in a Korean population-representative sample.

**Methods:**

A sample of 11,073 individuals (age 10–80 years) was included from the Sixth Korea National Health and Nutrition Examination Survey, 2014–2015. Isometric grip strength was assessed using a handheld dynamometer. Relative grip strength was calculated as the maximum absolute grip strength divided by body mass index. Means, standard error, and quintiles for grip strength and relative grip strength were analyzed per 5-year age group for each sex. To create growth charts for grip strength and relative grip strength, parametric quantile regression was used.

**Results:**

In males, absolute grip strength increased quickly starting from 10 years of age until approximately 20 years of age. In females, there was gradual growth until approximately 15 years of age. Grip strength continued to increase until a peak between ages 30 and 39 years, and then declined from midlife onwards in both sexes. Our data showed that the prevalence of weak grip strength increased rapidly in late adult life based on a T-score of -2 standard deviations below the sex-specific peak mean (equivalent to 33 kg in males and 20 kg in females). Approximately 20% of subjects had weak grip strength at age 65–69 years.

**Conclusion:**

This was the first study to establish normative reference values for grip strength across the lifespan in a Korean population aged 10–80 years. Percentiles of grip strength will inform clinical assessments and will help identify thresholds for the identification of muscle weakness.

## Introduction

Muscle weakness in older adults can affect functional limitations, morbidity, and mortality, even when it is not attributable to primary neurologic or muscular disease [1,2]. Muscle weakness is a key component of sarcopenia and the frailty phenotype in older adults [3,4] and is used as an indicator of malnutrition [5]. Furthermore, muscle weakness is also a primary marker of cardiovascular diseases and type 2 diabetes, as well as a predictor of all-cause death and cardiovascular death in middle-aged and young people [6–9]. Therefore, muscle weakness may act as a biomarker of healthy aging across the lifespan.

Grip strength measurements using a hand grip dynamometer are recommended for the assessment of muscle weakness. This is an easy, quick, noninvasive, and reliable measure of the maximum voluntary force of the hand. It is relatively inexpensive [10–12] and can be used in both clinical and community settings. This technique has been demonstrated to be a reliable screening tool in the assessment of nutrition risk during hospital admission [13,14]. Grip strength can also be informative with regard to nutritional status, muscle mass, physical function, and health status [13,15–17]. Thus, it should be routinely used as a vital sign throughout the life stages [18].

Reference values for grip strength are essential for making informed decisions about the normality of an individual’s status relative to the population. Published reference values for hand grip strength are available for many countries; in most cases, they are divided into age and sex subgroups [19–23]. Studies may divide participants by age, sex, right and left hand, or dominant and non-dominant hand [24]. Furthermore, reference values have been presented as the maximum grip strength measured for either hand [12,25–27].

More recently, normative reference values for grip strength have been published based on large, nationally representative Western populations using the U.S. National Health and Nutrition Examination Survey [26,28], the Canadian Health Measures Survey [27], the German Socio-Economic Panel [23], and 12 general population studies in Great Britain (U.K.) [29]. These nationally representative studies among Western populations covering the life course have reported that peak mean values of grip strength (around 48–54 kg for men and 28–34 kg for women) are not reached until the late forties, and they then decline from midlife onward in both sexes (mean values drop to about 41–44 kg for men and 25–28 kg for women). Moreover, the peak values reached in midlife among the German population are somewhat higher than those in the U.K., United States (U.S.), and Canada. Therefore, national variations in grip strength suggest that normative reference values developed for one country may not be applicable to other countries [30]; thus, reference values based on Western populations may not be appropriate for use in Asian populations. In particular, ethnicity should be taken into account because lifestyle and body composition are quite different between individuals who live in Western and Asian countries. To our knowledge, no previous studies have investigated the normative grip strength values in a study representative of Asian countries and covering the life course. However, some works have proposed normative grip strength values for specific age groups based on Asian populations [31–34]. A meta-analysis of previously published studies [31] among Japanese community-dwelling elderly adults who could independently perform activities of daily living estimated the mean grip strength of men aged 67–80 years as 33 kg and that of women aged 68–79 years as 21 kg. A pooled analysis of combined data from six cohorts of nondisabled, Japanese community-dwelling adults aged 65 years or older found mean grip strengths of 32 kg for men and 20 kg for women [32]. In other Asian countries, a cohort study among a nationally representative sample of Taiwanese adults aged 53 years and over reported average grip strengths of 34 kg for men and 20 kg for women [33]. Using a large sample dataset from Singapore, a representative Southeast Asian country [34], the normative grip strength values among community-dwelling elderly Singaporeans aged 60–89 years were defined: mean of 26 kg in men, mean of 17 kg in women. Typically, normative grip strength values for individuals of a given age and sex vary according to geographic region and/or ethnicity.

There is need to establish normative grip strength values based on a nationally representative study among a Korean population and covering the life course in Asian countries. These values would be of great help to health professionals and clinicians in clinical and epidemiologic settings. The primary aim of this analysis was to describe normative data for hand grip strength using a population-representative sample of Korean individuals aged 10–80 years from the Sixth Korea National Health and Nutrition Examination Survey (KNHANES VI), 2014–2015. A secondary analysis was performed to determine the relationship between hand grip strength and body size indicators. In a subanalysis, we sought to compare the mean values of maximum grip strength for Korean and U.S. populations, after stratification for sex, age, and body height.

## Materials and methods

### Study design and population

The KNHANES VI (2014–2015) is a nationwide, population-based, cross-sectional health examination and survey conducted by the Division of Chronic Disease Surveillance, Korea Centers for Disease Control and Prevention (KCDC), Ministry of Health and Welfare. KNHANES has been carried out annually since 1998 to monitor the health and nutrition status of the South Korean population [35]. KNHANES selects the subjects (by household units) using stratified, multistage probability sampling each year.

The KNHANES data are available to the public, having undergone a series of thorough reviews and cross-checking for missing information, erroneous values, mistaken codes, logical errors, missing values, and statistical outliers in the original data. We compiled all data of the 14,930 participants of the KNHANES VI. We initially selected 13,389 subjects aged ≥10 years for our study. Of the selected subjects, 11,104 subjects underwent grip strength examination. Subjects who were missing grip strength examinations that were tested in triplicate on each hand (*n* = 291) or who were missing height or weight data (*n* = 16) were excluded from our analyses. Moreover, we excluded 16 outliers, identified from specific regressions of model grip strength as a function of age and BMI in the linear regression; those with standardized residuals of more than ±3 SD were removed from the sample. Ultimately, 11,073 participants (5,054 men and 6,019 women) were included in our study.

All KNHANES surveys were approved by the Institutional Review Board (IRB) of the KCDC, and all the subjects provided written informed consent (IRB number: 2013-12EXP-03-5C). This study was exempt from IRB review by the Institutional Review Boards of Kyung Hee University (IRB number: KHSIRB-17-012).

### Anthropometric measurements

The subjects’ anthropometric measures were obtained by trained examiners. The height was determined to the nearest 0.1 cm using a wall-mounted stadiometer. Weight was measured with light clothing but without shoes to the nearest 0.1 kg. Waist circumference measurements were taken at the end of a normal expiration to the nearest 0.1 cm, measuring from the middle point between the lower border of the rib cage and the iliac crest at the mid-axillary line. Body mass index (BMI) was calculated as weight in kilograms divided by height in meters squared.

### Hand grip strength measurements

Grip strength examinations were performed as part of KNHANES VI (2014). All participants aged 10 and older were eligible for the hand grip strength component, which included a pretest questionnaire and an isometric grip strength test using a handheld Takei dynamometer (model TKK5401). Individuals were excluded if they reported hand or wrist surgery in the preceding 3 months or were unable to hold the dynamometer with either hand (e.g., paralyzed in both hands, missing limbs). Participants also were excluded if they had any pain, aching, or stiffness in their right hand in the past 7 days (e.g., arthritis, tendonitis, carpal tunnel syndrome).

When using the hand dynamometer, the subjects stood upright with the shoulders in a neutral position, arms at the side, and elbows fully extended. A trained examiner explained and demonstrated the protocol, adjusted the grip size for each hand, and asked the participant to squeeze the dynamometer for a practice trial. The handles were adjusted to accommodate hand size, with the index finger of each hand at 90° flexion between the proximal and middle interphalangeal joints. Then, the subjects were instructed to start the test by using each hand to squeeze the handle with maximum effort for 3 seconds, while being given verbal encouragement. Each hand was tested three times, alternating hands between trials with 60-second rests between measurements on the same hand. Absolute grip strength was calculated as the greatest reading for each hand and is expressed in kilograms (to the nearest 0.1 kg).

Hand grip strength is affected by changes in maturation and body size in children [20]. Previous studies have reported correlations between grip strength and anthropometric variables such as weight, height, and BMI among healthy people aged 18–90 years [36]. Therefore, grip strength has been expressed as a relative value. Several previous reports have indicated that hand grip strength is mostly dependent on body height and have suggested normative values stratified by body height [22,23,37,38]. Recently, using BMI to adjust for muscle strength has been recommended as a muscle quality index. For instance, the Foundation for the National Institutes of Health (FNIH) Sarcopenia Project has proposed a new approach for sarcopenia diagnosis in which muscle weakness is adjusted by BMI instead of body height or weight [39]. For the frailty phenotype among older adults, weakness has been defined, using maximum hand grip strength, as being at or below the 20th percentile within eight sex-by-BMI categories [3]. The relative grip strength-to-adjusted BMI ratio was associated with risk of limited mobility in older adults [40,41]. Moreover, hand grip strength divided by BMI was significantly associated with cardiometabolic risk, and the association was stronger than that using absolute hand grip strength [33]. Steffl et al. [42] suggested that the grip strength-to-BMI ratio could be used in children to diagnose sarcopenic obesity. Therefore, we calculated relative grip strength as the absolute grip strength divided by BMI. To enable inter study comparisons, both absolute and relative grip strengths are presented. In further analysis, we sought to compare the mean values of maximum grip strength for Korean and U.S. populations after stratification for sex, age, and body height. Furthermore, we aimed to compare mean vales reported in studies based on Western populations with normative values stratified by body height [22,23,37,38].

### Statistical analyses

The basic characteristics of the study population are reported as descriptive statistics, and all sample and weight variables have been stratified. To account for the complex survey design of stratified, random, and cluster sampling, we computed 2-year sample weights based on the methods recommended by the KCDC [43]. The means and standard errors (SE) of grip strength and relative grip strength were tabulated per 5-year age group for each sex. For males and females by age group, cutoff points for grip strength quintiles were calculated with SE estimates. Linear regression models with Bonferroni post hoc tests assessed age groups and sex differences.

To create growth charts for grip strength and relative grip strength, parametric quantile regression was used to determine the 5th, 10th, 20th, 25th, 50th, 75th, 80th, 90th, and 95th percentiles for ages 10–80 years. To explore the sex-specific growth patterns of normalized and absolute strength, parametric polynomial quantile regression models were used to predict strength with six powers of age (age^-1^, $\sqrt{age}$, $\mathrm{age}\sqrt{age}$, age, age^2^, age^3^) using the QUANTREG procedure in SAS version 9.3. Quantile regression was used for nine centiles to construct the strength growth charts for both male and female participants using the SAS macro, as described by Chen [44].

Based on the results of the secondary analysis, we defined a T-score for grip strength as an individual’s value expressed as a multiple of the number of standard deviations below the peak mean value encountered in young adult life. This is the same approach that is applied to bone density measurements in the diagnosis of osteoporosis [45], except we used sex-specific peak mean values for grip strength. We explored the sex-specific prevalence of weak grip strength in middle and late adult life using a T-score for grip strength that was equal to or less than -2 SD, as described previously [10]. To establish the correlations of grip strength with height, weight, body mass index, and waist circumference, complex samples general linear analyses were carried out with grip strength as a dependent variable and physical factors as independent variables. In a subanalysis, the mean values of maximum grip strength after stratification for sex, age, and height were tabulated for Korean and U.S. populations. Publicly available data of the National Health and Nutrition Examination Survey (NHANES 2013–2014) [46], which follows the same measurement protocol as KNHANES [47], were used for this analysis. NHANES is an ongoing series of cross-sectional surveys conducted by the National Center for Health Statistics (NCHS) of the U.S. Centers for Disease Control and Prevention (CDC). The NHANES sampling procedure oversamples targeted populations, such as Hispanics, non-Hispanic blacks, non-Hispanic Asians, older adults, and low-income individuals, to obtain adequate samples for meaningful subgroup analyses and more reliable variable estimates [48]. A total of 6,958 participants aged 10–80 years (3,395 men and 6,019 women) were included in our subanalysis.

We considered two-tailed *p* values of <0.05 to be statistically significant. Statistical analyses were performed using SAS version 9.3 (SAS Institute, Inc., Cary, NC) and the IBM SPSS Statistics 23 package (SPSS, Inc., Chicago, IL).

## Results

**Fig 1:**
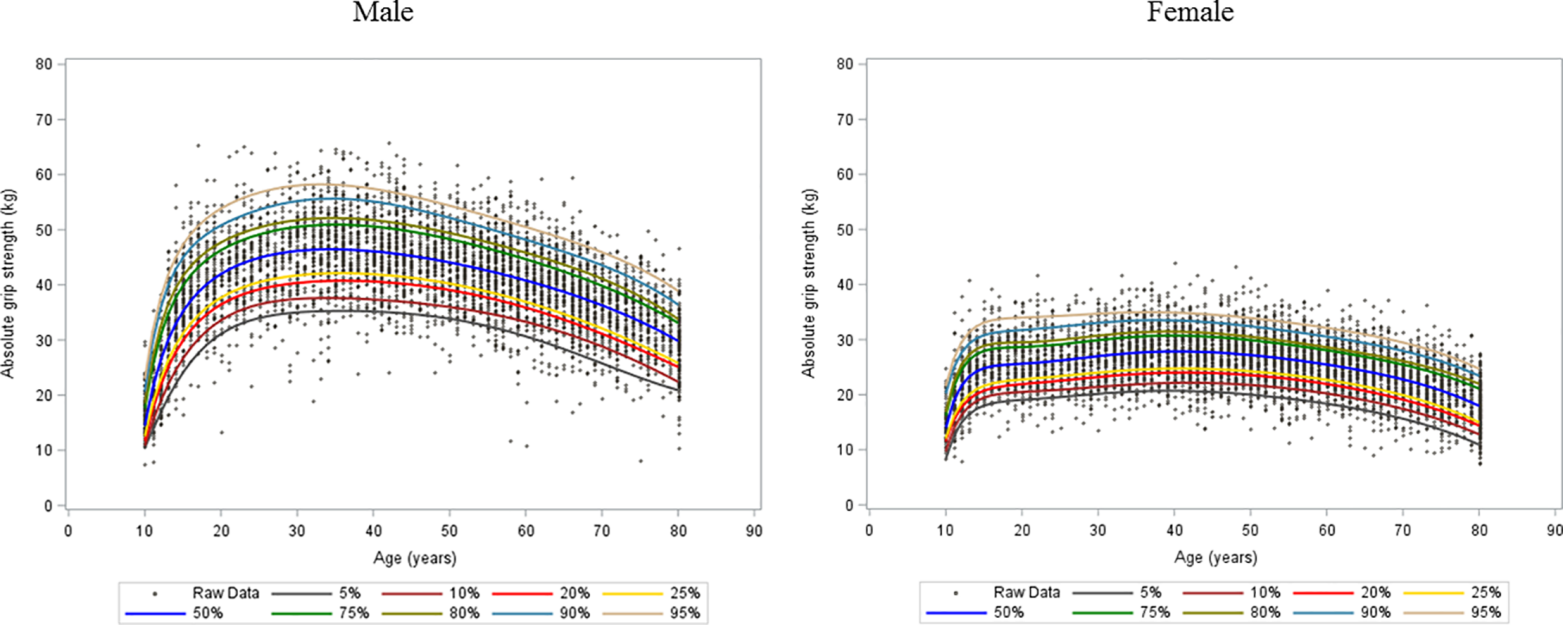
Absolute grip strength growth percentile curves for males and females.

**Fig 2:**
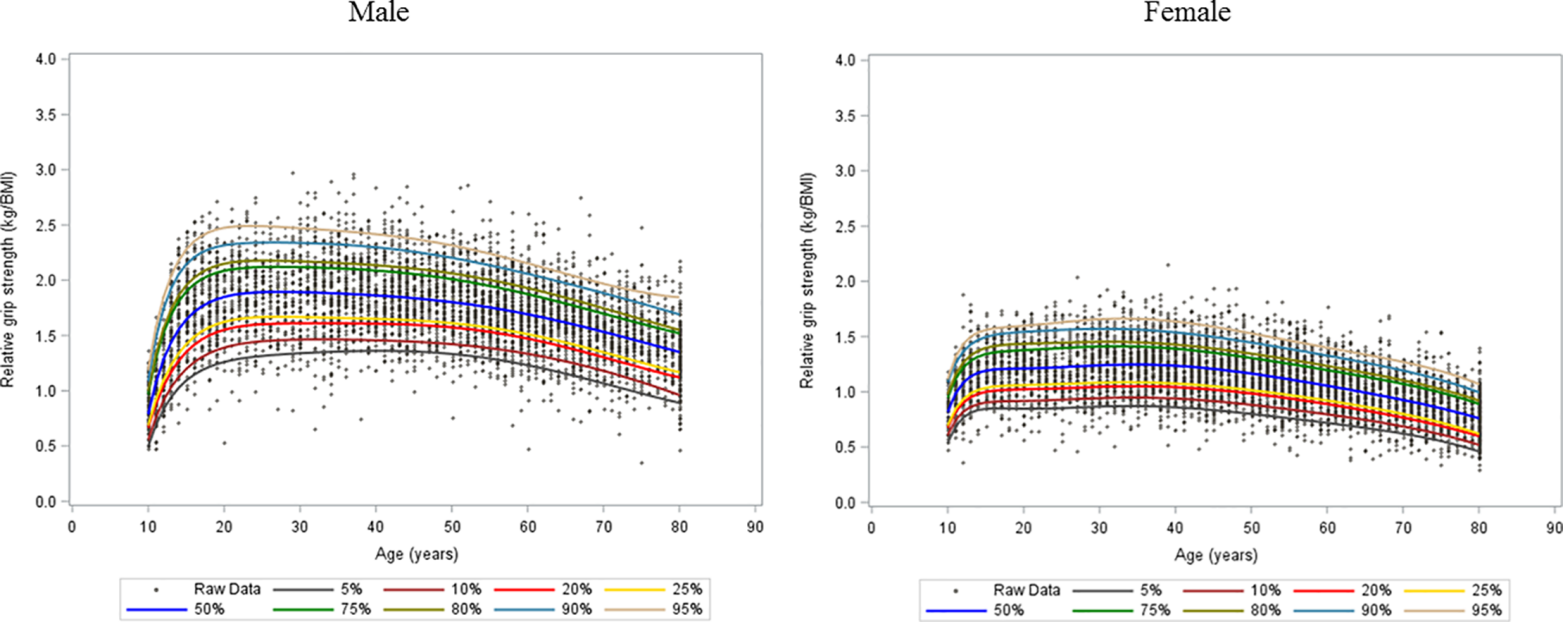
Relative grip strength growth percentile curves for males and females.

The mean age of the study population was 41.8 years (95% confidence interval [CI]: 41.2–42.3) for males and 44.1 years (95% CI: 43.4–44.8) for females. Mean BMI was 24.0 (95% CI: 23.9–24.01) for males and 22.9 (95% CI: 22.8–23.1) for females. The seven percentile curves together with the scatter plots of absolute grip strength and relative grip strength for males and females are shown in Figs 1 and 2. For males, absolute grip strength grew quickly from 10 years until approximately 20 years of age. Females showed gradual growth in absolute grip strength until approximately age 15 years. Grip strength continued to increase until a peak between ages 30 and 39, and then declined from midlife onward in both males and females. However, the rates of decline were greater among males. Males showed a higher peak of median absolute grip strength than females. Similar patterns of growth and decline can be observed in the relative grip strength curves in females. For males, there was quick growth until approximately age 20 years, with a slight decline from early adult life onward in both males and females.

The means, SEs, and quintiles for absolute grip strength and relative grip strength by sex and age group are shown in Tables 1 and 2. Our analysis of grip strength by sex showed a greater grip strength for males at all ages (P < 0.001). Males reached a peak median absolute grip strength of 47 kg and a peak relative grip strength of 1.90 between ages 30 and 39. For females, the peak median absolute grip strength was 28 kg and the peak relative grip strength was 1.26 between ages 35 and 39. In linear regression models, there was a decline from 45 years for males and 50 years for females, compared with peak median of grip strength (P < 0.001). For older individuals (age >70 years), the lowest quintile levels were 27.6 kg for males and 61.1 kg for females. The mean values for right-hand grip strength were higher than those for the left hand (P < 0.001). Dominant grip strength was similar to the right hand. Approximately 90% of the Korean population was right-hand dominant (Table 3). Dominant grip strength was higher than non-dominant hand in all age group (P < 0.001).

**Table 1:** Mean, standard error (SE), and quintiles for grip strength, by sex and age group (n = 11,073).

						Percentile				
Sex	Age group (years)	n	Mean	±	SE	20^th^	40^th^	50^th^	60^th^	80^th^
Male	10 to 14	413	24.9	±	0.5	16.6	20.1	22.4	25.2	33.9
	15 to 19	358	39.7	±	0.4	34.1	37.6	39.0	40.5	45.4
	20 to 24	249	42.5	±	0.5	37.2	40.0	42.3	44.4	47.7
	25 to 29	236	44.6	±	0.5	38.5	42.9	44.8	46.3	49.7
	30 to 34	317	46.7	±	0.5	41.4	45.0	47.0	49.0	52.5
	35 to 39	354	47.2	±	0.4	41.5	45.2	46.9	49.2	52.7
	40 to 44	406	46.3	±	0.4	40.6	44.4	45.9	47.4	52.0
	45 to 49	333	44.8	±	0.4	39.7	43.8	45.0	45.9	49.4
	50 to 54	386	43.3	±	0.3	38.1	41.7	43.0	44.8	48.1
	55 to 59	479	41.3	±	0.3	36.0	39.4	41.3	42.6	46.8
	60 to 64	394	40.1	±	0.4	35.4	38.3	40.1	41.3	45.6
	65 to 69	423	37.5	±	0.4	32.8	36.1	37.4	38.9	42.0
	70 to 74	312	34.7	±	0.4	29.9	33.3	34.7	36.8	38.8
	75 to 79	253	32.6	±	0.5	27.4	30.6	32.6	34.1	37.4
	80	148	28.6	±	0.5	24.0	27.5	29.3	30.4	32.3
	65 and above	1136	34.5	±	0.2	29.2	32.8	34.5	36.5	39.6
	70 and above	713	32.8	±	0.3	27.6	31.0	32.8	34.3	37.8
Female	10 to 14	340	20.6	±	0.3	16.1	19.2	20.0	21.4	24.9
	15 to 19	320	26.0	±	0.3	22.3	24.9	25.8	27.0	29.7
	20 to 24	322	25.9	±	0.3	21.7	24.0	25.4	26.7	29.7
	25 to 29	282	26.1	±	0.3	22.4	24.9	25.9	27.1	29.0
	30 to 34	420	27.5	±	0.3	23.1	26.4	27.2	28.6	31.0
	35 to 39	471	28.7	±	0.2	24.2	27.0	28.1	29.2	32.0
	40 to 44	512	27.9	±	0.2	23.9	26.4	27.5	28.6	31.0
	45 to 49	495	27.5	±	0.2	23.3	26.1	27.6	28.4	31.0
	50 to 54	535	26.9	±	0.2	23.2	25.9	27.0	27.9	30.0
	55 to 59	530	25.7	±	0.2	22.4	25.0	25.7	26.8	29.0
	60 to 64	476	25.0	±	0.2	21.6	24.1	25.5	26.3	28.1
	65 to 69	411	23.6	±	0.2	20.0	22.5	23.5	24.9	26.7
	70 to 74	383	22.0	±	0.3	18.5	20.9	22.0	23.3	25.6
	75 to 79	311	20.2	±	0.3	16.0	19.0	20.3	21.7	24.1
	80	211	17.4	±	0.3	13.7	16.2	17.2	18.2	21.1
	65 and above	1316	21.2	±	0.2	16.9	20.0	21.3	22.5	25.3
	70 and above	905	20.1	±	0.2	16.1	18.9	20.3	21.4	24.0

Weighted mean ± SE.

**Table 2:** Mean, standard error (SE), and quintiles for relative grip strength, by sex and age group (n = 11,073).

						Percentile				
Sex	Age group (years)	n	Mean	±	SE	20^th^	40^th^	50^th^	60^th^	80^th^
Male	10 to 14	413	1.22	±	0.02	0.87	1.06	1.14	1.24	1.55
	15 to 19	358	1.80	±	0.02	1.52	1.70	1.77	1.86	2.07
	20 to 24	249	1.86	±	0.02	1.56	1.77	1.84	1.95	2.14
	25 to 29	236	1.84	±	0.02	1.56	1.77	1.86	1.94	2.11
	30 to 34	317	1.90	±	0.02	1.61	1.83	1.90	1.98	2.17
	35 to 39	354	1.87	±	0.02	1.64	1.85	1.90	2.02	2.19
	40 to 44	406	1.85	±	0.02	1.61	1.75	1.85	1.92	2.12
	45 to 49	333	1.78	±	0.02	1.62	1.78	1.86	1.94	2.08
	50 to 54	386	1.72	±	0.01	1.53	1.70	1.76	1.86	2.00
	55 to 59	479	1.67	±	0.02	1.47	1.63	1.72	1.78	1.97
	60 to 64	394	1.72	±	0.01	1.45	1.59	1.67	1.74	1.88
	65 to 69	423	1.58	±	0.02	1.36	1.51	1.58	1.63	1.79
	70 to 74	312	1.48	±	0.02	1.27	1.41	1.47	1.54	1.68
	75 to 79	253	1.42	±	0.02	1.19	1.35	1.41	1.49	1.63
	80	148	1.29	±	0.03	1.02	1.21	1.28	1.35	1.51
	65 and above	1136	1.48	±	0.01	1.24	1.40	1.47	1.55	1.72
	70 and above	713	1.42	±	0.01	1.18	1.35	1.41	1.48	1.65
Female	10 to 14	340	1.05	±	0.01	0.86	0.98	1.05	1.10	1.25
	15 to 19	320	1.22	±	0.02	1.00	1.14	1.21	1.27	1.43
	20 to 24	322	1.23	±	0.01	1.05	1.18	1.21	1.27	1.43
	25 to 29	282	1.21	±	0.01	0.99	1.14	1.20	1.25	1.43
	30 to 34	420	1.25	±	0.01	1.02	1.17	1.24	1.32	1.46
	35 to 39	471	1.26	±	0.01	1.07	1.19	1.26	1.31	1.46
	40 to 44	512	1.22	±	0.01	1.02	1.15	1.23	1.28	1.41
	45 to 49	495	1.19	±	0.01	1.00	1.14	1.19	1.24	1.36
	50 to 54	535	1.14	±	0.01	0.97	1.08	1.14	1.20	1.31
	55 to 59	530	1.10	±	0.01	0.91	1.04	1.10	1.14	1.29
	60 to 64	476	1.04	±	0.01	0.86	0.99	1.04	1.09	1.14
	65 to 69	411	0.98	±	0.01	0.80	0.91	0.97	1.02	1.15
	70 to 74	383	0.91	±	0.01	0.74	0.85	0.90	0.95	1.08
	75 to 79	311	0.83	±	0.01	0.67	0.76	0.82	0.89	0.99
	80	211	0.75	±	0.02	0.57	0.68	0.73	0.80	0.90
	65 and above	1316	0.88	±	0.01	0.69	0.82	0.87	0.93	1.06
	70 and above	905	0.84	±	0.01	0.65	0.78	0.83	0.89	1.01

Weighted mean ± SE. Relative grip strength = the absolute grip strength divided by body mass index.

**Table 3:** Mean, standard error (SE), lowest quintile of dominant-hand, non-dominant-hand, right-hand and left-hand Grip Strength (kg) by sex and age groups.

		Dominant-hand^a^				Non-dominant-hand^b^				Right-hand^c^				Left-hand^d^			
Sex	Age group (years)	Mean	±	SE	Lowest quintile	Mean	±	SE	Lowest quintile	Mean	±	SE	Lowest quintile	Mean	±	SE	Lowest quintile
Male	10 to 14	24.5	±	0.5	16.5	23.1	±	0.4	15.4	24.4	±	0.5	16.5	23.2	±	0.4	15.6
	15 to 19	39.2	±	0.4	33.8	36.9	±	0.4	31.8	39.0	±	0.4	33.7	37.1	±	0.4	31.8
	20 to 24	42.2	±	0.5	36.9	39.9	±	0.5	34.6	42.1	±	0.5	36.8	40.0	±	0.5	34.6
	25 to 29	44.2	±	0.5	38.2	41.3	±	0.5	35.6	44.0	±	0.5	37.9	41.7	±	0.5	35.9
	30 to 34	46.5	±	0.5	41.0	43.7	±	0.5	38.3	46.3	±	0.5	40.6	43.8	±	0.5	38.4
	35 to 39	46.9	±	0.5	40.7	44.4	±	0.4	38.6	46.8	±	0.5	40.3	44.5	±	0.5	38.5
	40 to 44	45.9	±	0.4	40.3	43.4	±	0.4	37.7	45.7	±	0.4	40.1	43.5	±	0.4	37.8
	45 to 49	44.2	±	0.4	39.0	42.3	±	0.4	37.2	44.1	±	0.4	38.6	42.4	±	0.4	37.4
	50 to 54	42.7	±	0.3	37.3	40.9	±	0.4	35.2	42.6	±	0.3	37.4	40.9	±	0.4	35.0
	55 to 59	40.5	±	0.4	34.5	38.9	±	0.3	33.5	40.3	±	0.4	34.3	39.2	±	0.3	34.1
	60 to 64	39.4	±	0.4	33.9	37.7	±	0.4	32.6	39.3	±	0.4	33.8	37.7	±	0.3	32.6
	65 to 69	36.8	±	0.4	31.9	35.2	±	0.4	29.7	36.8	±	0.3	32.1	35.1	±	0.4	29.7
	70 to 74	34.0	±	0.4	29.3	32.3	±	0.4	27.6	33.9	±	0.3	28.9	32.5	±	0.4	27.7
	75 to 79	31.5	±	0.5	26.3	30.2	±	0.5	25.5	31.4	±	0.5	26.1	30.7	±	0.5	25.8
	80	27.7	±	0.5	22.3	26.5	±	0.5	22.4	27.4	±	0.5	22.1	26.3	±	0.5	21.9
	65 and above	33.6	±	0.2	28.1	32.3	±	0.2	27.0	33.6	±	0.2	28.1	32.2	±	0.2	26.8
	70 and above	31.9	±	0.3	26.3	30.5	±	0.3	25.4	31.7	±	0.3	26.3	30.6	±	0.3	25.4
Female	10 to 14	20.3	±	0.3	15.8	19.1	±	0.3	14.9	20.3	±	0.3	15.8	19.1	±	0.3	14.9
	15 to 19	25.7	±	0.3	22.0	24.0	±	0.3	20.0	25.6	±	0.3	21.9	24.1	±	0.3	20.1
	20 to 24	25.7	±	0.3	21.6	24.0	±	0.3	20.1	25.6	±	0.3	21.6	24.1	±	0.3	20.4
	25 to 29	25.9	±	0.3	21.9	23.9	±	0.3	20.1	25.8	±	0.3	21.9	23.9	±	0.3	20.1
	30 to 34	27.2	±	0.3	22.7	25.1	±	0.3	21.5	27.2	±	0.3	22.7	25.3	±	0.3	21.6
	35 to 39	27.9	±	0.3	24.1	26.2	±	0.2	22.2	27.8	±	0.2	23.8	26.4	±	0.2	22.5
	40 to 44	27.2	±	0.2	23.5	25.8	±	0.2	22.1	27.1	±	0.2	23.4	25.8	±	0.2	22.2
	45 to 49	27.2	±	0.3	22.9	25.2	±	0.3	21.2	27.1	±	0.2	22.9	25.3	±	0.2	21.3
	50 to 54	26.4	±	0.2	22.9	24.9	±	0.2	21.3	26.4	±	0.2	22.9	24.9	±	0.2	21.0
	55 to 59	25.3	±	0.2	21.9	23.7	±	0.2	20.3	25.3	±	0.2	21.9	23.6	±	0.2	20.1
	60 to 64	24.6	±	0.2	21.3	23.0	±	0.2	20.0	24.6	±	0.2	21.2	23.1	±	0.2	20.0
	65 to 69	23.2	±	0.3	19.3	21.5	±	0.2	18.3	23.2	±	0.3	19.4	21.6	±	0.2	18.1
	70 to 74	21.5	±	0.2	18.2	20.1	±	0.3	16.6	21.6	±	0.2	18.2	20.1	±	0.3	16.5
	75 to 79	19.8	±	0.3	15.7	18.6	±	0.3	14.9	19.8	±	0.3	15.5	18.7	±	0.3	15.1
	80	16.9	±	0.4	13.0	15.8	±	0.3	12.3	16.9	±	0.3	13.0	15.8	±	0.3	12.4
	65 and above	20.8	±	0.2	16.4	19.4	±	0.2	15.4	20.7	±	0.2	16.3	19.4	±	0.2	15.5
	70 and above	19.7	±	0.2	15.5	18.4	±	0.2	14.6	19.7	±	0.2	15.6	18.5	±	0.2	14.5

Weighted mean ± SE.

^a^n = 11407

^b^n = 10693 (exclusion of both dominant hands)

^c^n = 11395

^d^n = 11392

**Fig 3:**
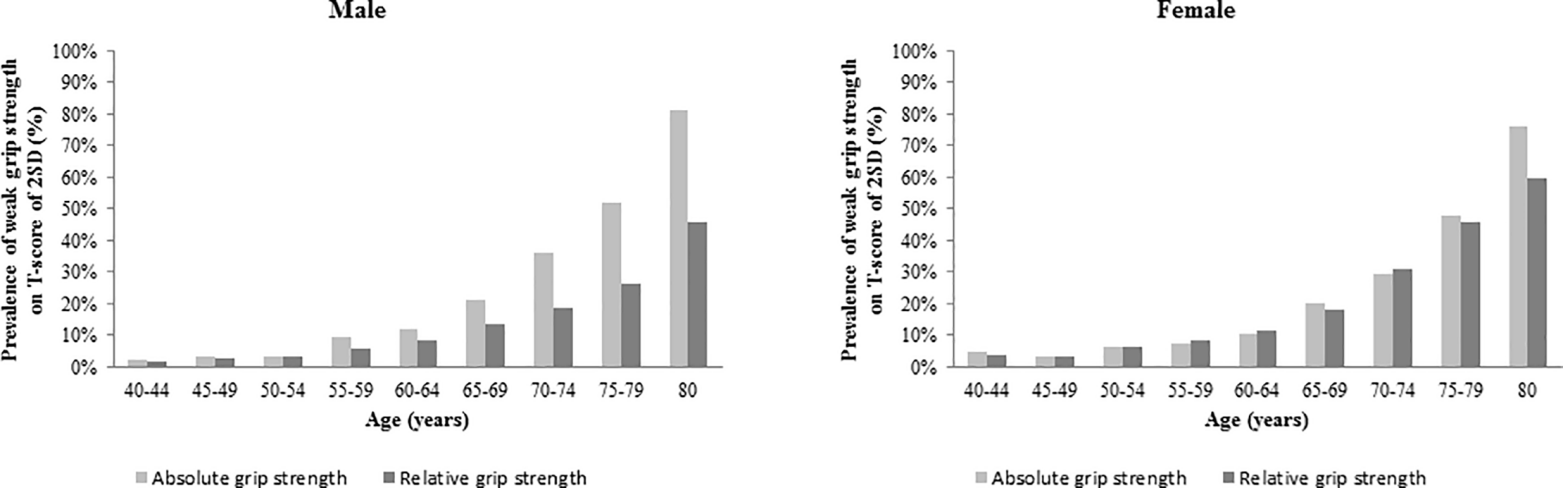
Sex-specific prevalence of weak grip strength based on T-scores of -2 SD sex-specific norms in young adults.

The estimated prevalence rates of weak grip strength in middle and late adult life are shown in Fig 3. Weak grip strength was defined as a gender-specific T-score being ≤-2 SD. These values are relative to the peak mean (SD) scores for absolute and relative grip strength of 47.1 (6.9) kg and 1.92 (0.33) kg in males and 28.1 (4.3) kg and 1.26 (0.23) kg in females, respectively; both occurred between the ages of 35 and 39 years (S1 Table). Males and females had a similar prevalence of weak grip strength during the decline phase. The prevalence of weak grip increased rapidly in late adult life. Our results showed that approximately 21% of men and 20% of women had weak absolute grip strength at age 65. In men, the weakness was more prevalent in absolute grip strength than in relative grip strength.

S2 Table shows results obtained for the correlation between hand grip strength and physical factors (height, weight, BMI, and waist circumference). The coefficients of determination (r^2^) of these correlations were 0.338, 0.310, and 0.138 for grip strength, height, weight, and BMI in men and 0.238, 0.152, 0.026 in women, respectively (all *p* <0.001). The mean values of maximum grip strength after stratification for sex, age, and height for the Korean and U.S. populations are shown in S3 and S4 Tables. For Korean males, the normative value for the age group 35–39 years with body height 165–169 cm is 45 kg. This value increases by about 2 kg for every 5 cm of additional body height, up to 179 cm. By contrast, the normative value for the age group 35–39 years of the same body height in U.S. populations is 46 kg, and this value increases by about 3 kg for every 5 cm of additional body height. For Korean females in the same age group, we found that mean values of grip strength increase by 1–2 kg for each 5 cm of body height. For females in the U.S., the mean value of grip strength increases by 1–3 kg for each 5 cm of body height.

## Discussion

Our study established nationally representative estimates of muscular grip strength over the lifespan for the Korean population aged 10–80 years. Our normative values for grip strength across the lifespan will aid in the clinical assessment of grip strength, to help establish thresholds for the identification of muscle weakness and to inform consensus definitions of sarcopenia and frailty in older populations.

Our study is the first to produce normative data for grip strength across the whole lifespan using a population-representative sample from an Asian population. We found that normative absolute grip strength followed a similar pattern across the lifespan as in Western populations, increasing to a peak in early and middle adult life, plateauing in midlife, and declining from midlife onward [23,26,27,29]. Compared with normative data from Western populations, the peak median values in our study tended to be somewhat lower for absolute grip strength (47 kg for men and 28 kg for women). Normative data collected from 12 British studies identified peak median grip strength values of 51 kg for males between ages 29 and 39 years and 31 kg for females between ages 26 and 42 years [29]. These results are close to the normative values from the U.S. NHANES by Peterson and Krishnan [26]. The Canadian Health Measures Survey [27], using reference equations for Canadians aged 6–79 years, reported median maximum grip strength values of 48.4 kg for males and 28.5 kg for females between ages 40 and 44 years. German Socio-Economic Panel [23] representative data for the German population showed that pooled means for men are about 54 kg (ages 30–49 years) and about 34.5 kg (ages 35–44 years). The differences in the growth curves for grip strength may be partly owing to the effects of body size. Our study found a strong positive association between grip strength and body height in Korean populations. Previous studies of Western populations determined normative values after stratifying for sex, age, and body height [22,23,37]. In other reports, normative vales for grip strength were determined using the large UK Biobank dataset, which includes 502,713 people from across the general population in the U.K. aged 39–73 years [22], as well as data from three large nationwide population-based surveys among Danish populations aged 45–102 years [37]. Different average grip strengths for participants of the same height (165–169.9 cm) and age (45–49 years) were found in the abovementioned British (40 kg for males and 28 kg for females) and Danish studies (50 kg for males and 31 kg for females). The reported values for a German study [23] (50 kg for men and 34 kg for women) were similar to those of the Danish study for men.

The methods used to characterize grip strength may vary based on the choice of dynamometer or the measurement protocol. Our previous work demonstrated statistically significant differences in grip strength according to dynamometer type and whether measurements were taken in a seated or standing position [49]. In an additional analysis, our study aimed to compare the mean values of maximum grip strength for Korean and U.S. populations, after stratifying for sex, age, and body height, using the same measurement protocol as the KNHANES for grip strength. We confirmed age- and sex-specific grip strength in the U.S. sample, assuming they had the same body height as their Korean counterparts. For instance, the normative value for Korean men aged 45–49 years with a body height of 165–169 cm is about 43 kg, and 46 kg for those who are 10 cm taller. By contrast, in a U.S. population, the normative values for men aged 45–49 years with body height 165–169 cm are about 45 kg (50 kg for those 175–179 cm tall). To take account of this difference in age- and sex-specific grip strength, a 2 to 4 kg difference in the same height group between the Korean and U.S. populations. There are several possible explanations for the difference in normative data for grip strength between regions and ethnic backgrounds, including differences in body size and composition, such as mean height and weight. To our knowledge, no previous works have determined the normative grip strength values in a nationally representative study of Asian countries covering the life course. Here, we were unable to compare Asian populations, as we did not investigate age- and sex- stratified height (and BMI) alongside grip strength. However, some studies have proposed normative grip strength values for older adults based on Asian populations [31–34]. Malhotra et al. [34] reported normative grip strength values among community-dwelling elderly Singaporeans aged 60–89 years (means, 26 kg in men and 17 kg in women for the dominant hand). Seino et al. [32] reported data from six cohorts among Japanese community-dwelling adults aged 65 years or older (means, 32 kg for men and 20 kg for women for the dominant hand). We found that elderly Korean men are somewhat stronger than their counterparts in other older Asian populations, such as in Japan and Singapore.

Age-related declines in grip strength were shown to start as early as the fifth decade of life. The life course trajectory identified for muscle weakness and any cutoff values related to relevant health outcomes were important. We examined the sex-specific prevalence of weak grip strength in middle and late adult life with a T-score for grip strength that was ≤-2 SD, as used previously by Lauretani et al [10]. Our study found that the prevalence of weak grip strength increased rapidly in late adult life based on a T-score of ≤-2 SD (equivalent to 33 kg in males and 20 kg in females), with approximately 20% of participants having weak grip strength at age 65–69 years in both sexes. Our study used similar cutoff values as other studies. Twelve British studies demonstrated a high prevalence of weak grip strength (-2 SD: 32 kg in males and 19 kg in females), with almost half of participants scoring at or below this level by age 80 years and a prevalence of 20% at age 70 years [30]. A German study using data from five waves of the German Socio-Economic Panel [23] defined muscle weakness by values that were 2 SD below the sex-specific peak means, with a weak grip being defined to start below 33 kg for males and below 21 kg for females. Moreover, those studies suggested the threshold to define critically weak grip associated with elevated mortality risk, using a cut-off at 1 SD below the peak means (44 kg for men and 28 kg for women). In this study, by ages 60–64 years for men and 65–69 years for women, the average grip strength has dropped by 1 SD (to 40 kg for men and to 24 kg for women). By contrast, the muscle weakness cut-points for Canadians (–2 SD: 27.9 kg in men and 16.4 kg in women) were inconsistent with the findings of our study. In addition, a lower prevalence of weak grip strength was found for Canadians, ranging from 3–5% among 3,181 respondents aged 60–79 years from the 2007–2013 Canadian Health Measures Survey [50]. These T-score differences are likely attributable to the size of standard deviation differences based on grip strength among a sample of young Canadians adults (e.g., mean of 48.5 kg [SD 10.3] for men aged 20 to 39 years).

In older populations, muscle weakness is a key component of sarcopenia and the frailty phenotype [3,4]. It is also regarded as an indicator of malnutrition [5]. Several grip strength cutoff values to identify clinically relevant weakness and frailty phenotypes have been proposed by the Cardiovascular Health Study [3], the European Working Group on Sarcopenia in Older People [4], the FNIH Sarcopenia Project [51], and the Asian Working Group for Sarcopenia (AWGS) [52]. Cutoff values for weakness were defined in the 2014 FNIH Sarcopenia Project (26 kg for males and 16 kg for females) and AWGS (26 kg for males and 18 kg for females). Our data showed that the lowest quintile of maximum grip strength for age 70 years or older was approximately 28 kg for males and 16 kg for females, whereas the lowest quintile for dominant-hand grip strength was approximately 26 kg for males and 16 kg for females (Table 3). Our findings can help to confirm relationships in longitudinal trajectories of weak grip strength when individual values are used to determine risk. In particular, age- and sex-based grip strength criteria that are specific to sarcopenia or frailty in a Korean population should be defined in the future.

This study is representative of a large Korean population, which is a major strength. However, this study also has some limitations. First, our data for the reference equations developed in this study apply only to people age 10–80 years who had their data collected using similar dynamometers and test protocols. Second, our normative data for grip strength are cross-sectional and are likely to underestimate individual decline. Third, the analyzed normative data were obtained from both healthy and unhealthy populations, based on a survey sample representative of the entire non-institutionalized general population of Korea. Further research into the reference values among healthy individuals without chronic conditions that can influence grip strength is required [27]. Furthermore, the inference of exposure thresholds or percentiles ascertained from cross-sectional data make it difficult to understand longitudinal changes or the direction of causation between exposure and the outcomes of interest. Therefore, future studies are needed to establish the clinical relevance and prognostic value of our results. Finally, this study calculated relative grip strength as the absolute grip strength divided by BMI because normalized strength is associated with sarcopenia [26].

## Conclusions

We established nationally representative estimates of muscular grip strength over the lifespan. Our data will be useful for the development of grip strength reference equations for Korean populations 10–80 years of age. The percentiles of grip strength were compared to populations from other countries to identify normative values and were constructed to include both age- and sex-specific absolute and relative grip strength growth curves. These normative reference values for grip strength across the lifespan can inform the clinical assessment of grip strength. Our findings also will help to establish thresholds for the identification of muscle weakness in a consensus definition of sarcopenia and frailty among older populations.

## Supporting information

S1 Table Unweighted mean and standard deviations (SD) for maximal grip strength and relative grip strength, by sex and age for a population from KNHANES VI. (DOCX)

S2 Table Linear regression of grip strength on anthropometric characteristics, by sex. (DOCX)

S3 Table Unweighted means for maximal grip strength by sex, age, and body height, for a population from KNHANES VI. (DOCX)

S4 Table Unweighted means for maximal grip strength by sex, age, and body height, from a U.S. representative sample. (DOCX)

S1 File Data set files used for the study analysis. (XLSX)
