# Supplementary material for: Muscular grip strength normative values for a Korean population from the Korea National Health and Nutrition Examination Survey, 2014–2015
Source: PLoS One. 2018 Aug 20;13(8):e0201275. doi: 10.1371/journal.pone.0201275 (PMC6101358; doi:10.1371/journal.pone.0201275)
Supplement: S1 Table — (DOCX) [file pone.0201275.s001.docx]

**S1 Table.** Unweighted mean and standard deviations (SD) for maximal grip strength and

relative grip strength, by sex and age for a population from KNHANES VI (n=11,073)

|  |  | Male | | |  | Female | | |
| --- | --- | --- | --- | --- | --- | --- | --- | --- |
|  | Age group (years) | Mean | ± | SD |  | Mean | ± | SD |
| Maximal grip strength | 10 to 14 | 24.9 | ± | 9.0 |  | 20.5 | ± | 5.3 |
|  | 15 to 19 | 39.5 | ± | 6.9 |  | 26.1 | ± | 4.4 |
|  | 20 to 24 | 42.8 | ± | 7.2 |  | 25.9 | ± | 4.8 |
|  | 25 to 29 | 44.4 | ± | 7.0 |  | 25.9 | ± | 4.2 |
|  | 30 to 34 | 46.6 | ± | 7.4 |  | 27.5 | ± | 4.4 |
|  | 35 to 39 | 47.1 | ± | 6.9 |  | 28.1 | ± | 4.3 |
|  | 40 to 44 | 46.3 | ± | 6.7 |  | 27.6 | ± | 4.4 |
|  | 45 to 49 | 44.6 | ± | 6.0 |  | 27.5 | ± | 4.3 |
|  | 50 to 54 | 43.2 | ± | 5.9 |  | 26.8 | ± | 4.3 |
|  | 55 to 59 | 41.5 | ± | 6.4 |  | 25.9 | ± | 4.0 |
|  | 60 to 64 | 40.2 | ± | 6.4 |  | 25.1 | ± | 3.9 |
|  | 65 to 69 | 37.6 | ± | 6.1 |  | 23.6 | ± | 4.4 |
|  | 70 to 74 | 34.8 | ± | 5.4 |  | 22.0 | ± | 4.1 |
|  | 75 to 79 | 32.8 | ± | 5.9 |  | 20.2 | ± | 4.3 |
|  | 80 | 28.5 | ± | 5.7 |  | 17.6 | ± | 4.0 |
| Maximal relative  grip strength^a^ | 10 to 14 | 1.22 | ± | 0.40 |  | 1.05 | ± | 0.24 |
|  | 15 to 19 | 1.79 | ± | 0.35 |  | 1.21 | ± | 0.23 |
|  | 20 to 24 | 1.86 | ± | 0.36 |  | 1.23 | ± | 0.23 |
|  | 25 to 29 | 1.84 | ± | 0.32 |  | 1.20 | ± | 0.24 |
|  | 30 to 34 | 1.89 | ± | 0.36 |  | 1.26 | ± | 0.24 |
|  | 35 to 39 | 1.92 | ± | 0.33 |  | 1.26 | ± | 0.23 |
|  | 40 to 44 | 1.86 | ± | 0.31 |  | 1.22 | ± | 0.22 |
|  | 45 to 49 | 1.84 | ± | 0.29 |  | 1.20 | ± | 0.23 |
|  | 50 to 54 | 1.78 | ± | 0.30 |  | 1.14 | ± | 0.21 |
|  | 55 to 59 | 1.72 | ± | 0.29 |  | 1.09 | ± | 0.22 |
|  | 60 to 64 | 1.68 | ± | 0.29 |  | 1.03 | ± | 0.19 |
|  | 65 to 69 | 1.57 | ± | 0.27 |  | 0.98 | ± | 0.21 |
|  | 70 to 74 | 1.49 | ± | 0.27 |  | 0.91 | ± | 0.19 |
|  | 75 to 79 | 1.43 | ± | 0.27 |  | 0.83 | ± | 0.19 |
|  | 80 | 1.29 | ± | 0.30 |  | 0.76 | ± | 0.19 |

Unweighted mean ± SD.
