# Supplementary material for: Muscular grip strength normative values for a Korean population from the Korea National Health and Nutrition Examination Survey, 2014–2015
Source: PLoS One. 2018 Aug 20;13(8):e0201275. doi: 10.1371/journal.pone.0201275 (PMC6101358; doi:10.1371/journal.pone.0201275)
Supplement: S2 Table — (DOCX) [file pone.0201275.s002.docx]

**S2 Table.** Linear regression of grip strength on anthropometric characteristics, by sex for a population from

KNHANES VI

|  | Male | | | | Female | | | |
| --- | --- | --- | --- | --- | --- | --- | --- | --- |
|  | β | SE | 95% CI | R^2^ | β | SE | 95% CI | R^2^ |
| Height (cm) | 0.65 | 0.02 | 0.62, 0.68^**^ | 0.338 | 0.37 | 0.01 | 0.35, 0.40^**^ | 0.238 |
| Weight (kg) | 0.38 | 0.01 | 0.35, 0.40^**^ | 0.310 | 0.21 | 0.01 | 0.19, 0.23^**^ | 0.152 |
| Waist circumference (cm) | 0.30 | 0.02 | 0.27, 0.33^**^ | 0.118 | 0.05 | 0.01 | 0.03, 0.07^**^ | 0.009 |
| Body mass index (kg/m^2^) | 0.91 | 0.04 | 0.83, 1.00^**^ | 0.138 | 0.23 | 0.03 | 0.18, 0.28^**^ | 0.026 |
| < 18.5 | -10.61 | 0.71 | -12.01, -9.21^**^ | 0.132 | -2.90 | 0.28 | -3.45, -2.34^**^ | 0.028 |
| 18.5-24.9 | *Ref.* |  |  |  | *Ref.* |  |  |  |
| ≥ 25.0 | 3.56 | 0.27 | 3.03, 4.09^**^ |  | 0.45 | 0.19 | 0.09, 0.82^*^ |  |

Complex samples general linear analyses

* P < 0.05, ** P < 0.001

β, standardized coefficients; CI, confidence intervals; R^2^,coefficient of determination, SE, standard error
