# Supplementary material for: Muscular grip strength normative values for a Korean population from the Korea National Health and Nutrition Examination Survey, 2014–2015
Source: PLoS One. 2018 Aug 20;13(8):e0201275. doi: 10.1371/journal.pone.0201275 (PMC6101358; doi:10.1371/journal.pone.0201275)
Supplement: S3 Table — (DOCX) [file pone.0201275.s003.docx]

**S3 Table.** Unweighted means for maximal grip strength by sex, age, and body height, for a population

from KNHANES VI

| **Age, y** | **Height, cm** | **Mean GS**  **for Male, kg** | **Mean GS**  **for Female, kg** | **Age, y** | **Height, cm** | **Mean GS**  **for Male, kg** | **Mean GS**  **for Female, kg** |
| --- | --- | --- | --- | --- | --- | --- | --- |
| 15 to 19 | < 140 | — | — | 50 to 54 | < 140 | — | — |
|  | 140 -149.9 | — | — |  | 140 -149.9 | — | 23.1 ± 3.8 |
|  | 150 -154.9 | — | 24.1 ± 3.9 |  | 150 -154.9 | — | 25.9 ± 4.0 |
|  | 155 -159.9 | — | 25.8 ± 4.1 |  | 155 -159.9 | 41.9 ± 5.6 | 27.0 ± 3.9 |
|  | 160 -164.9 | 37.4 ± 5.9 | 25.6 ± 4.3 |  | 160 -164.9 | 41.1 ± 5.1 | 27.5 ± 4.4 |
|  | 165 -169.9 | 38.1 ± 6.0 | 28.3 ± 4.7 |  | 165 -169.9 | 43.0 ± 6.0 | 30.0 ± 4.0 |
|  | 170 -174.9 | 39.5 ± 6.4 | 29.9 ± 4.2 |  | 170 -174.9 | 43.6 ± 5.4 | 34.5 ± 2.1 |
|  | 175 -179.9 | 40.7 ± 7.4 | — |  | 175 -179.9 | 45.2 ± 5.8 | — |
|  | ≥ 180 | 42.6 ± 7.0 | — |  | ≥ 180 | 49.6 ± 8.1 | — |
| 20 to 24 | < 140 | — | — | 55 to 59 | < 140 | — | — |
|  | 140 -149.9 | — | — |  | 140 -149.9 | — | 23.9 ± 4.1 |
|  | 150 -154.9 | — | 24.2 ± 4.0 |  | 150 -154.9 | — | 25.1 ± 3.9 |
|  | 155 -159.9 | — | 25.2 ± 4.7 |  | 155 -159.9 | 38.5 ± 5.9 | 25.9 ± 3.9 |
|  | 160 -164.9 | 36.2 ± 10 | 25.6 ± 4.7 |  | 160 -164.9 | 39.8 ± 6.1 | 27.9 ± 4.1 |
|  | 165 -169.9 | 39.6 ± 7.8 | 27.2 ± 3.8 |  | 165 -169.9 | 40.8 ± 6.7 | 28.5 ± 3.4 |
|  | 170 -174.9 | 42.6 ± 7.0 | 28.4 ± 5.9 |  | 170 -174.9 | 43.3 ± 5.6 | — |
|  | 175 -179.9 | 45.1 ± 6.7 | 32.4 ± 4.1 |  | 175 -179.9 | 42.9 ± 5.3 | — |
|  | ≥ 180 | 43.5 ± 5.6 | - |  | ≥ 180 | 47.7 ± 7.9 | — |
| 25 to 29 | < 140 | — | — | 60 to 64 | < 140 | — | — |
|  | 140 -149.9 | — | — |  | 140 -149.9 | — | 23.2 ± 3.7 |
|  | 150 -154.9 | — | 23.9 ± 3.4 |  | 150 -154.9 | — | 24.7 ± 4.2 |
|  | 155 -159.9 | — | 24.7 ± 4.4 |  | 155 -159.9 | — | 25.6 ± 3.6 |
|  | 160 -164.9 | 39.5 ± 10.0 | 26.4 ± 4.0 |  | 160 -164.9 | 38.2 ± 6.5 | 26.9 ± 4.0 |
|  | 165 -169.9 | 43.1 ± 7.5 | 27.0 ± 4.1 |  | 165 -169.9 | 40.3 ± 6.1 | — |
|  | 170 -174.9 | 45.0 ± 5.8 | 27.2 ± 4.7 |  | 170 -174.9 | 43.1 ± 6.1 | — |
|  | 175 -179.9 | 45.9 ± 6.4 | — |  | 175 -179.9 | 42.9 ± 5.3 | — |
|  | ≥ 180 | 44.5 ± 9.0 | — |  | ≥ 180 | — | — |
| 30 to 34 | < 140 | — | — | 65 to 69 | < 140 | — | — |
|  | 140 -149.9 | — | — |  | 140 -149.9 | — | 20.8 ± 4.0 |
|  | 150 -154.9 | — | 25.3 ± 4.6 |  | 150 -154.9 | — | 23.4 ± 4.1 |
|  | 155 -159.9 | — | 26.4 ± 4.3 |  | 155 -159.9 | 34.2 ± 5.4 | 24.6 ± 4.0 |
|  | 160 -164.9 | — | 27.9 ± 4.3 |  | 160 -164.9 | 35.9 ± 5.3 | 26.4 ± 4.5 |
|  | 165 -169.9 | 44.9 ± 7.6 | 29.2 ± 4.1 |  | 165 -169.9 | 38.3 ± 5.9 | — |
|  | 170 -174.9 | 45.4 ± 7.6 | 29.4 ± 3.9 |  | 170 -174.9 | 40.1 ± 6.0 | — |
|  | 175 -179.9 | 47.5 ± 7.1 | 33.2 ± 3.3 |  | 175 -179.9 | 42.0 ± 5.2 | — |
|  | ≥ 180 | 49.1 ± 6.5 | - |  | ≥ 180 | — | — |
| 35 to 39 | < 140 | — | — | 70 to 74 | < 140 | — | — |
|  | 140 -149.9 | — | — |  | 140 -149.9 | — | 20.6 ± 3.6 |
|  | 150 -154.9 | — | 25.8 ± 3.2 |  | 150 -154.9 | — | 22.3 ± 3.9 |
|  | 155 -159.9 | — | 27.8 ± 4.2 |  | 155 -159.9 | 33.0 ± 4.9 | 23.6 ± 4.4 |
|  | 160 -164.9 | 44.0 ± 5.8 | 28.5 ± 4.4 |  | 160 -164.9 | 34.4 ± 4.8 | 22.3 ± 4.2 |
|  | 165 -169.9 | 44.6 ± 5.1 | 29.7 ± 4.1 |  | 165 -169.9 | 35.3 ± 5.9 | — |
|  | 170 -174.9 | 47.0 ± 6.5 | 30.4 ± 4.2 |  | 170 -174.9 | 36.4 ± 5.9 | — |
|  | 175 -179.9 | 48.7 ± 6.8 | — |  | 175 -179.9 | 34.4 ± 4.4 | — |
|  | ≥ 180 | 50.3 ± 7.0 | — |  | ≥ 180 | — | — |
| 40 to 44 | < 140 | — | — | 75 to 79 | < 140 | — | 18.6 ± 4.1 |
|  | 140 -149.9 | — | 24.9 ± 4.5 |  | 140 -149.9 | — | 19.3 ± 4.0 |
|  | 150 -154.9 | — | 25.9 ± 4.3 |  | 150 -154.9 | 29.1 ± 5.0 | 20.4 ± 4.1 |
|  | 155 -159.9 | — | 27.0 ± 4.2 |  | 155 -159.9 | 30.3 ± 4.1 | 21.7 ± 4.9 |
|  | 160 -164.9 | 41.0 ± 6.1 | 28.7 ± 4.2 |  | 160 -164.9 | 32.7 ± 6.3 | 21.3 ± 4.8 |
|  | 165 -169.9 | 44.6 ± 5.9 | 28.6 ± 4.4 |  | 165 -169.9 | 33.4 ± 6.0 | — |
|  | 170 -174.9 | 46.3 ± 6.1 | 30.0 ± 6.2 |  | 170 -174.9 | 34.9 ± 5.7 | — |
|  | 175 -179.9 | 48.3 ± 6.4 | — |  | 175 -179.9 | 38.3 ± 4.5 | — |
|  | ≥ 180 | 48.7 ± 6.5 | — |  | ≥ 180 | — | — |
| 45 to 49 | < 140 | — | — | 80 | < 140 | — | 15.8 ± 3.1 |
|  | 140 -149.9 | — | 25.1 ± 3.2 |  | 140 -149.9 | — | 17.4 ± 4.0 |
|  | 150 -154.9 | — | 26.3 ± 3.7 |  | 150 -154.9 | 27.9 ± 5.0 | 18.4 ± 4.5 |
|  | 155 -159.9 | — | 27.1 ± 4.0 |  | 155 -159.9 | 26.6 ± 5.2 | 19.1 ± 3.3 |
|  | 160 -164.9 | 41.6 ± 6.0 | 28.4 ± 4.3 |  | 160 -164.9 | 28.0 ± 5.2 | — |
|  | 165 -169.9 | 43.2 ± 5.6 | 30.3 ± 5.5 |  | 165 -169.9 | 30.9 ± 5.3 | — |
|  | 170 -174.9 | 45.7 ± 6.0 | — |  | 170 -174.9 | 33.7 ± 5.8 | — |
|  | 175 -179.9 | 45.8 ± 4.9 | — |  | 175 -179.9 | — | — |
|  | ≥ 180 | 47.4 ± 7.0 | — |  | ≥ 180 | — | — |

Unweighted mean ± SD; —10 or fewer individuals
