# Supplementary material for: Muscular grip strength normative values for a Korean population from the Korea National Health and Nutrition Examination Survey, 2014–2015
Source: PLoS One. 2018 Aug 20;13(8):e0201275. doi: 10.1371/journal.pone.0201275 (PMC6101358; doi:10.1371/journal.pone.0201275)
Supplement: S4 Table — (DOCX) [file pone.0201275.s004.docx]

**S4 Table.** Unweighted means for maximal grip strength by sex, age, and body height, from a U.S.

representative sample^a^

| **Age, y** | **Height, cm** | **Mean GS**  **for Male, kg** | **Mean GS**  **for Female, kg** | **Age, y** | **Height, cm** | **Mean GS**  **for Male, kg** | **Mean GS**  **for Female, kg** |
| --- | --- | --- | --- | --- | --- | --- | --- |
| 15 to 19 | < 140 | — | — | 50 to 54 | < 140 | — | — |
|  | 140 -149.9 | — | — |  | 140 -149.9 | — | — |
|  | 150 -154.9 | — | 27.7 ± 3.9 |  | 150 -154.9 | — | 27.2 ± 4.3 |
|  | 155 -159.9 | 36.8 ± 5.0 | 28.6 ± 4.6 |  | 155 -159.9 | — | 28.4 ± 4.5 |
|  | 160 -164.9 | 37.3 ± 6.9 | 30.0 ± 4.9 |  | 160 -164.9 | 39.9 ± 6.3 | 31.1 ± 4.7 |
|  | 165 -169.9 | 40.5 ± 7.1 | 31.3 ± 5.3 |  | 165 -169.9 | 43.4 ± 8.4 | 35.6 ± 5.3 |
|  | 170 -174.9 | 44.9 ± 6.9 | 34.4 ± 6.4 |  | 170 -174.9 | 44.7 ± 6.8 | 31.7 ± 7.7 |
|  | 175 -179.9 | 45.3 ± 7.9 | — |  | 175 -179.9 | 46.8 ± 6.5 | — |
|  | ≥ 180 | 49.3 ± 8.5 | — |  | ≥ 180 | 51.8 ± 7.0 | — |
| 20 to 24 | < 140 | — | — | 55 to 59 | < 140 | — | — |
|  | 140 -149.9 | — | — |  | 140 -149.9 | — | 23.9 ± 5.2 |
|  | 150 -154.9 | — | 28.9 ± 4.7 |  | 150 -154.9 | — | 26.0 ± 4.6 |
|  | 155 -159.9 | — | 29.8 ± 4.5 |  | 155 -159.9 | — | 27.3 ± 5.0 |
|  | 160 -164.9 | — | 32.0 ± 5.0 |  | 160 -164.9 | 39.2 ± 8.1 | 29.4 ± 5.4 |
|  | 165 -169.9 | 43.8 ± 7.9 | 33.1 ± 4.9 |  | 165 -169.9 | 41.8 ± 4.9 | 32.2 ± 5.6 |
|  | 170 -174.9 | 46.6 ± 7.6 | 34.0 ± 3.6 |  | 170 -174.9 | 46.2 ± 6.5 | 32.2 ± 7.0 |
|  | 175 -179.9 | 49.3 ± 10.6 | — |  | 175 -179.9 | 44.8 ± 7.7 | — |
|  | ≥ 180 | 53.7 ± 9.2 | — |  | ≥ 180 | 48.1 ± 6.8 | — |
| 25 to 29 | < 140 | — | — | 60 to 64 | < 140 | — | — |
|  | 140 -149.9 | — | — |  | 140 -149.9 | — | 21.8 ± 3.7 |
|  | 150 -154.9 | — | 28.6 ± 5.1 |  | 150 -154.9 | — | 26.0 ± 5.4 |
|  | 155 -159.9 | — | 30.0 ± 3.5 |  | 155 -159.9 | 32.1 ± 5.4 | 25.2 ± 5.1 |
|  | 160 -164.9 | 41.0 ± 7.5 | 31.9 ± 5.5 |  | 160 -164.9 | 35.8 ± 6.7 | 28.9 ± 4.8 |
|  | 165 -169.9 | 46.4 ± 6.6 | 34.4 ± 5.7 |  | 165 -169.9 | 39.7 ± 6.1 | 30.0 ± 4.9 |
|  | 170 -174.9 | 47.2 ± 9.1 | 32.6 ± 32.6 |  | 170 -174.9 | 41.0 ± 7.0 | — |
|  | 175 -179.9 | 51.1 ± 9.2 | — |  | 175 -179.9 | 42.1 ± 8.7 | — |
|  | ≥ 180 | 52.4 ± 8.7 | — |  | ≥ 180 | 49.6 ± 8.2 | — |
| 30 to 34 | < 140 | — | — | 65 to 69 | < 140 | — | — |
|  | 140 -149.9 | — | 26.9 ± 4.2 |  | 140 -149.9 | — | 23.0 ± 4.7 |
|  | 150 -154.9 | — | 27.7 ± 4.0 |  | 150 -154.9 | — | 25.3 ± 4.2 |
|  | 155 -159.9 | — | 30.5 ± 4.5 |  | 155 -159.9 | — | 25.1 ± 4.3 |
|  | 160 -164.9 | 44.7 ± 7.1 | 32.7 ± 6.0 |  | 160 -164.9 | 35.9 ± 7.9 | 27.4 ± 5.9 |
|  | 165 -169.9 | 45.9 ± 8.8 | 33.3 ± 5.0 |  | 165 -169.9 | 38.5 ± 6.3 | 29.0 ± 5.4 |
|  | 170 -174.9 | 49.3 ± 7.8 | 34.6 ± 4.8 |  | 170 -174.9 | 43.6 ± 7.0 | — |
|  | 175 -179.9 | 51.6 ± 7.3 | — |  | 175 -179.9 | 43.0 ± 6.2 | — |
|  | ≥ 180 | 54.5 ± 8.0 | — |  | ≥ 180 | 45.4 ± 8.6 | — |
| 35 to 39 | < 140 | — | — | 70 to 74 | < 140 | — | — |
|  | 140 -149.9 | — | 26.7 ± 3.2 |  | 140 -149.9 | — | 20.3 ± 3.8 |
|  | 150 -154.9 | — | 28.7 ± 4.2 |  | 150 -154.9 | — | 23.4 ± 4.3 |
|  | 155 -159.9 | — | 30.5 ± 4.6 |  | 155 -159.9 | — | 23.4 ± 4.7 |
|  | 160 -164.9 | — | 32.2 ± 5.2 |  | 160 -164.9 | 31.7 ± 4.3 | 26.1 ± 4.3 |
|  | 165 -169.9 | 45.7 ± 6.3 | 34.5 ± 6.3 |  | 165 -169.9 | 38.8 ± 6.8 | 26.4 ± 3.8 |
|  | 170 -174.9 | 48.7 ± 8.0 | 37.2 ± 6.0 |  | 170 -174.9 | 38.7 ± 6.2 | — |
|  | 175 -179.9 | 52.0 ± 8.9 | — |  | 175 -179.9 | 41.6 ± 7.4 | — |
|  | ≥ 180 | 54.1 ± 8.7 | — |  | ≥ 180 | 46.7 ± 6.7 | — |
| 40 to 44 | < 140 | — | — | 75 to 79 | < 140 | — | — |
|  | 140 -149.9 | — | 26.9 ± 3.4 |  | 140 -149.9 | — | 19.3 ± 3.6 |
|  | 150 -154.9 | — | 28.3 ± 4.2 |  | 150 -154.9 | — | 21.8 ± 4.7 |
|  | 155 -159.9 | — | 29.4 ± 4.9 |  | 155 -159.9 | — | 21.9 ± 4.6 |
|  | 160 -164.9 | 41.4 ± 5.0 | 32.1 ± 6.0 |  | 160 -164.9 | 32.4 ± 3.3 | 23.8 ± 4.5 |
|  | 165 -169.9 | 46.5 ± 6.1 | 33.0 ± 5.3 |  | 165 -169.9 | 33.4 ± 9.6 | 26.4 ± 4.8 |
|  | 170 -174.9 | 47.7 ± 6.7 | 35.0 ± 4.7 |  | 170 -174.9 | 35.6 ± 6.2 | — |
|  | 175 -179.9 | 51.1 ± 6.5 | — |  | 175 -179.9 | 38.6 ± 7.2 | — |
|  | ≥ 180 | 54.2 ± 8.2 | — |  | ≥ 180 | 40.9 ± 8.3 | — |
| 45 to 49 | < 140 | — | — | 80 | < 140 | — | — |
|  | 140 -149.9 | — | — |  | 140 -149.9 | — | 17.1 ± 4.7 |
|  | 150 -154.9 | — | 28.1 ± 4.9 |  | 150 -154.9 | — | 20.2 ± 5.1 |
|  | 155 -159.9 | — | 31.2 ± 5.2 |  | 155 -159.9 | — | 21.5 ± 4.4 |
|  | 160 -164.9 | — | 31.1 ± 4.6 |  | 160 -164.9 | 29.3 ± 5.4 | 21.0 ± 5.0 |
|  | 165 -169.9 | 45.2 ± 7.7 | 31.8 ± 5.2 |  | 165 -169.9 | 30.6 ± 5.9 | 20.6 ± 6.0 |
|  | 170 -174.9 | 48.2 ± 8.3 | 34.8 ± 5.1 |  | 170 -174.9 | 33.2 ± 6.7 | — |
|  | 175 -179.9 | 49.9 ± 5.6 | — |  | 175 -179.9 | 33.8 ± 6.9 | — |
|  | ≥ 180 | 51.5 ± 7.8 | — |  | ≥ 180 | 34.9 ± 6.7 | — |

^a^ The 2013-2014 National Health and Nutrition Examination Survey; n = 6,958 (male; n= 3,395, female; n= 3,563)

Unweighted mean ± SD; —10 or fewer individuals
